# Molecular evolution and phylogenetic relationships of *Ligusticum* (Apiaceae) inferred from the whole plastome sequences

**DOI:** 10.1186/s12862-022-02010-z

**Published:** 2022-04-30

**Authors:** Ting Ren, Dengfeng Xie, Chang Peng, Lingjian Gui, Megan Price, Songdong Zhou, Xingjin He

**Affiliations:** 1 Key Laboratory of Bio-Resources and Eco-Environment of Ministry of Education, College of Life Sciences, Sichuan University, Chengdu, 610065 China; 2 Sichuan Key Laboratory of Conservation Biology on Endangered Wildlife, College of Life Sciences, Sichuan University, Chengdu, 610065 China

**Keywords:** *Ligusticum*, Plastome, Molecular evolution, Concatenation, Coalescent, Phylogenomics

## Abstract

**Background:**

The genus *Ligusticum* belongs to Apiaceae, and its taxonomy has long been a major difficulty. A robust phylogenetic tree is the basis of accurate taxonomic classification of *Ligusticum*. We herein used 26 (including 14 newly sequenced) plastome-scale data to generate reliable phylogenetic trees to explore the phylogenetic relationships of Chinese *Ligusticum*.

**Results:**

We found that these plastid genomes exhibited diverse plastome characteristics across all four currently identified clades in China, while the plastid protein-coding genes were conserved. The phylogenetic analyses by the concatenation and coalescent methods obtained a more robust molecular phylogeny than prior studies and showed the non-monophyly of Chinese *Ligusticum*. In the concatenation-based phylogeny analyses, the two datasets yielded slightly different topologies that may be primarily due to the discrepancy in the number of variable sites.

**Conclusions:**

Our plastid phylogenomics analyses emphasized that the current circumscription of the Chinese *Ligusticum* should be reduced, and the taxonomy of *Ligusticum* urgently needs revision. Wider taxon sampling including the related species of *Ligusticum* will be necessary to explore the phylogenetic relationships of this genus. Overall, our study provided new insights into the taxonomic classification of *Ligusticum* and would serve as a framework for future studies on taxonomy and delimitation of *Ligusticum* from the perspective of the plastid genome.

**Supplementary Information:**

The online version contains supplementary material available at 10.1186/s12862-022-02010-z.

## Background

*Ligusticum* L., belonging to the Apiaceae family, has long been known for its medicinal values. The Chinese pharmacopeia [1] records that the dried rhizomes or roots of *L. sinense* Oliv. or *L. jeholense* Nakai et Kitag. can dispel wind, disperse cold, remove dampness, and relieve pain, and thus can be used for wild-cold, parietal headache, and rheumatism arthralgia. In addition, the essential oil and supercritical fluid (SFE-CO_2_) extract of *L. pteridophyllum* Franch. rhizome have significantly insecticidal properties, and for this reason, can be developed as a more environmentally benignant insecticide [2]. The *Ligusticum* genus has a broad circumscription where it comprises 40–60 species and is distributed predominantly in Asia, Europe, and North America [3–5]. Forty *Ligusticum* species have been identified (35 species are endemic) in China with most inhabiting the alpine and subalpine belt of Southwestern China, and only a few species distributed in the mountainous areas of Northern China [5, 6].

*Ligusticum* is one of the most complex genera in Apiaceae, and the taxonomy remains uncertain [5], resulting largely from the diversity of flowers, leaves, bracteoles, and mericarps [5, 7, 8]. Its relationships with allied genera *Cnidium*, *Hymenidium*, *Pachypleurum*, *Paraligusticum*, *Rupiphila*, *Selinum*, *Tilingia*, and *Ligusticopsis* are still not elucidated clearly [5]. *Ligusticum* has long been of interest to many plant taxonomists and numerous studies have been reported, such as on pollen morphology [9], karyological studies [10], cladistic analysis [11], leaf epidermal morphology [12], fruit features [13], and molecular phylogeny [8, 14]. Early years ago, Pu [6] mainly focused on bracteoles, in conjunction with fruits and palynological characters to divide *Ligusticum* into two sections: *L.* section *Ligusticum* L. and *L.* section *Pinnatibracteola* Pu. Yet this split has not been adopted by other scholars and is not reflected in later molecular phylogeny [8, 15, 16]. Many molecular phylogenetic studies have implied the non-monophyly of *Ligusticum* [14–21], and recent studies identified six clades within *Ligusticum*: *Acronema* Clade, *Conioselinum chinense* Clade, Pyramidoptereae, Selineae, *Sinodielsia* Clade, and *East-Asia* Clade [8]. So far, all molecular phylogenetic analyses are based on smaller datasets (a single or a few genes), except for Ren et al. [16] using plastome-scale datasets. Nevertheless, few *Ligusticum* species were involved in this plastid phylogenomics study. Hence, a greater taxon sampling is indispensable to confirm the phylogenetic position of *Ligusticum*.

Next-generation sequencing technology provides more DNA sequencing data than before and can be employed for phylogenetic studies within angiosperms [22]. Meanwhile, plastome-scale data has been used successfully to address phylogenetic problems at various taxonomic levels. For example, Li et al. [23] used 2881  plastid genomes to construct angiosperm phylogeny and date the origin of the crown angiosperms to the Upper Triassic. Wang et al. [24] constructed the phylogeny of *Angelica* and demonstrated the power of plastid phylogenomics in resolving the phylogeny of this complex genus. Wen et al. [25] revealed a new backbone relationship of Apioideae from plastid phylogenomic analysis. At present, there are two major methods to construct phylogenetic trees: the concatenation method and the coalescent method. Generally, the coalescent method can construct a phylogenetic tree more accurately than the concatenation method, and the concatenation method may produce spuriously high bootstrap support but topologically incorrect phylogenetic trees with the addition of more data [26, 27]. Recent studies have shown that it is necessary to construct the phylogenetic tree of plastid protein-coding genes by the coalescent method [28–30]. Hence, we utilized these two methods to estimate the phylogeny of *Ligusticum*.

Here, 26 *Ligusticum* plastomes (including 14 newly sequenced) representing all four currently identified clades in China were used for molecular evolutionary analysis and phylogenetic reconstruction. Our aims were to (1) describe the diversity of plastome characteristics and the evolutionary pattern of plastid protein-coding genes within *Ligusticum*; (2) obtain a robust *Ligusticum* phylogeny and assess the power of the plastome-scale data for resolving the phylogeny of this genus; (3) comment on the current taxonomy of *Ligusticum* in China based on the plastome sequences.

## Results

### Features of *Ligusticum* plastomes

**Fig. 1:**
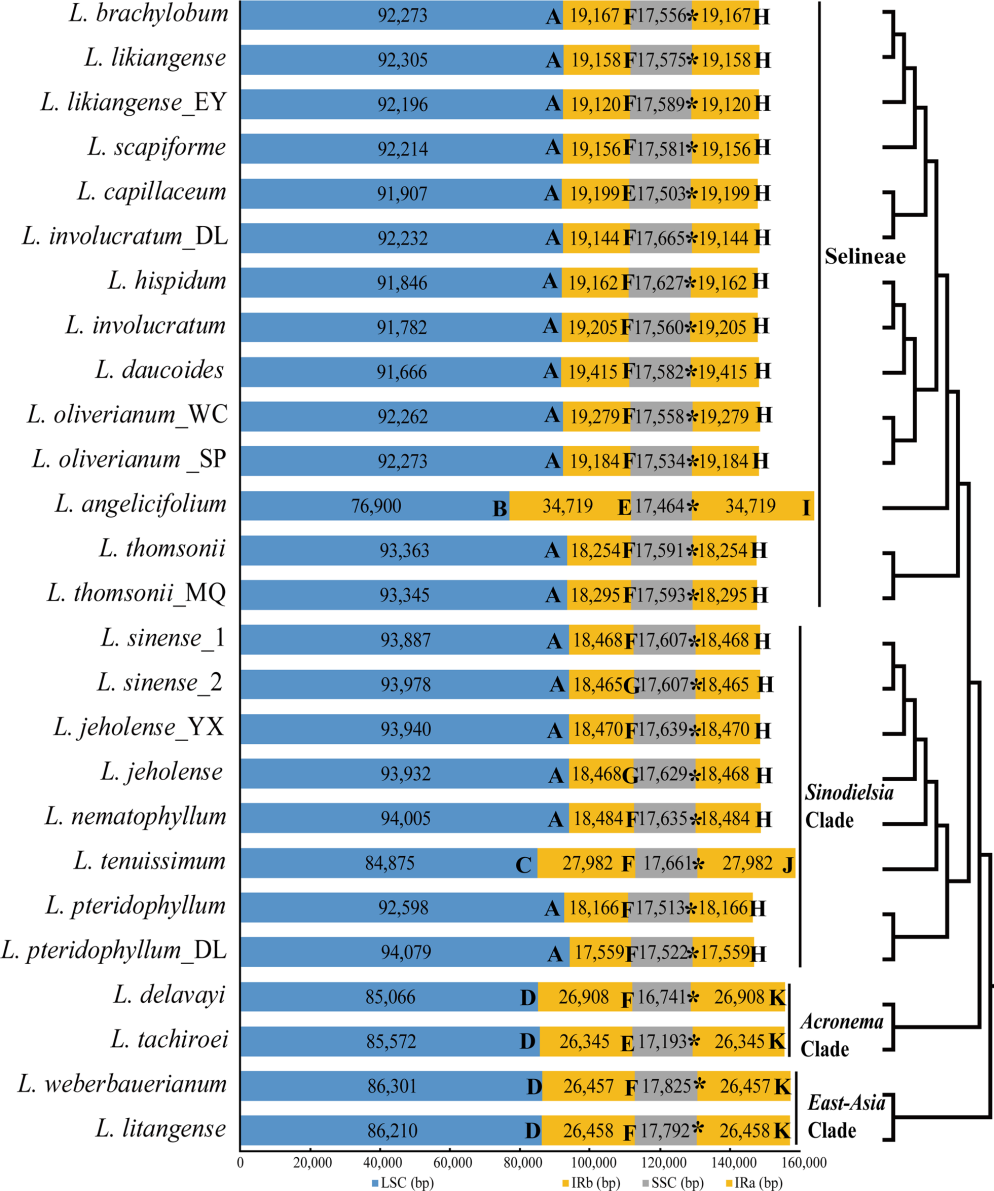
The total length stacked bar chart of 26 *Ligusticum* plastomes composed of four regions (LSC, IRb, SSC, and IRa). The numbers on the bar represent the length of the four regions. A–K Represents the genes at IR/SC borders. A *ycf2*; B *petB*; C *rpl22*; D *rps19*; E *ycf1/ndhF*; F *ycf1*; G *trnN-GUU-ndhF*; H *trnL-CAA-trnH-GUG*; I *petB-trnH-GUG*; J *rps19-trnH-GUG*; K *rpl2-trnH-GUG*. All the SSC/IRa borders are *ycf1*, which is indicated by asterisks

**Fig. 2:**
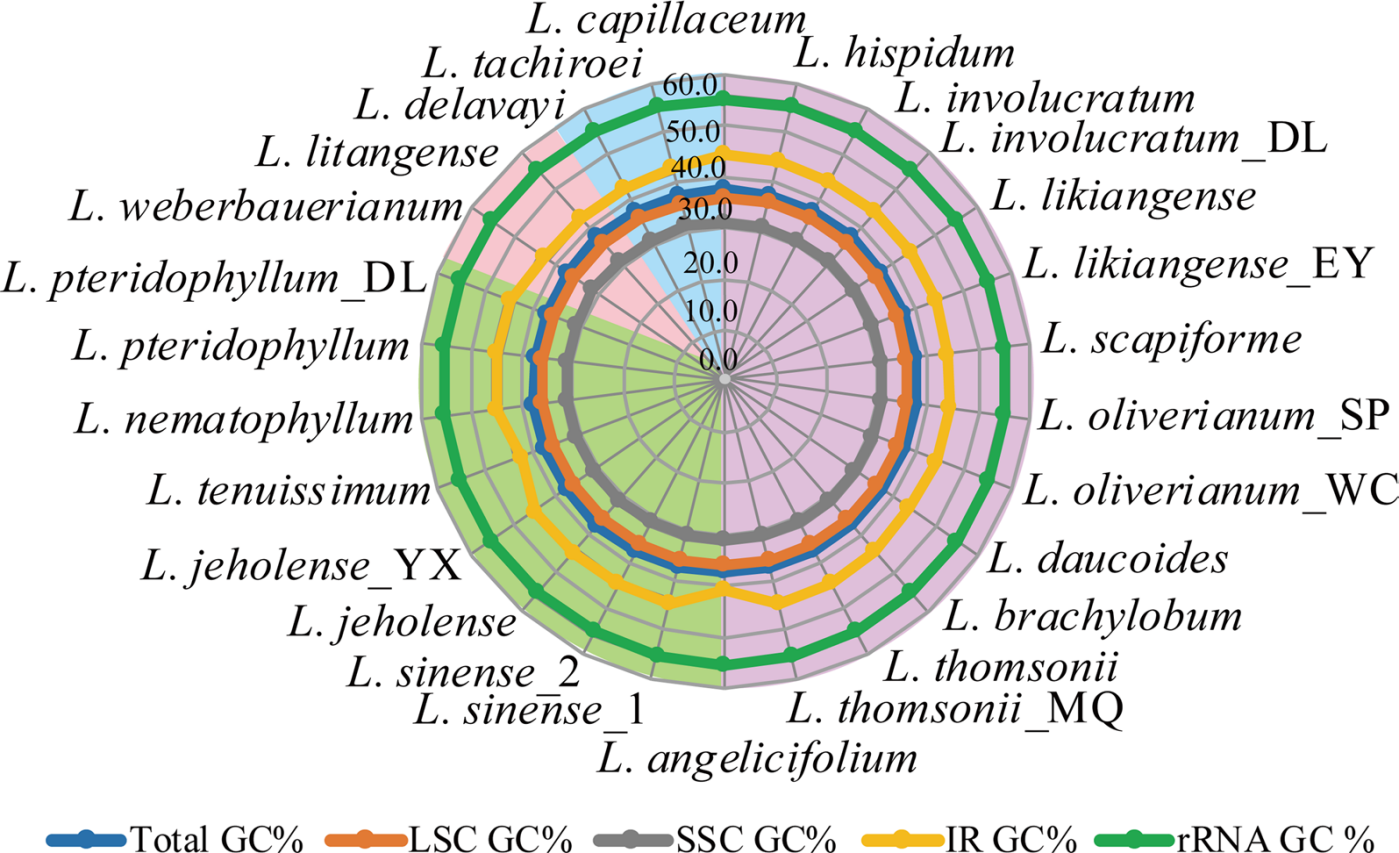
Comparison of the GC content (GC%) of 26 *Ligusticum* plastomes using a radar-plot. From inside to out: SSC GC%, LSC GC%, Total GC%, IR GC%, and rRNA GC%. The background colors of purple, green, blue, and pink represent Selineae, *Sinodielsia* Clade, *Acronema* Clade, and *East-Asia* Clade, respectively

The Illumina NovaSeq sequencing yielded between 32,046,626 (*L. tachiroei*) and 50,722,538 (*L. litangense*) clean reads from the 14 newly sequenced species, with the mean base coverage ranging from 278× (*L. jeholense*_YX) to 1950× (*L. nematophyllum*) (Table 1). Among the four clades, the *Ligusticum* plastomes were variable (Table 1, Fig. 1). Selineae and *Sinodielsia* Clade had similar plastome sizes and IR/SC borders. The total sequence length varied from 146,443 bp (*L. pteridophyllum*) to 148,608 bp (*L. nematophyllum*) except for *L. tenuissimum* (158,500 bp) and *L. angelicifolium* (163,802 bp). The IR/SC borders were the same except for the IR/LSC borders of the above two plastomes. For *Acronema* Clade and *East-Asia* Clade, the plastome sizes and the IR/SC borders were highly similar. The total sequence length varied from 155,455 bp (*L. tachiroei*) to 157,040 bp (*L. weberbauerianum*), and the IR/SC borders were identical except for the slightly different IRb/SSC border found in *L. tachiroei*. Among these 26 plastomes, *L. angelicifolium* had the longest plastome length (163,802 bp), which is caused by the significant expansion of IR regions (34,719 bp) (Table 1, Fig. 1, Additional file 1: Fig. S1). The LSC/IRb border extended into the *petB* gene and the IRa/LSC border extended into *petB-trnH-GUG* in this plastome, whereas the LSC/IRb border extended into *ycf2*, *rpl22*, or *rps19* gene, and the IRa/LSC border extended into *trnL-CAA-trnH-GUG*, *rps19-trnH-GUG*, or *rpl2-trnH-GUG* for the other *Ligusticum* species (Fig. 1). All *Ligusticum* plastomes possessed 128–145 genes, comprising 84–100 protein-coding genes, 36–37 tRNA genes, and eight rRNA genes (Table 1, Additional file 1: Fig. S1). *Ligusticum* species among the four clades possessed nearly identical GC content not only in whole plastome (37.4–37.6%) but also in LSC (35.7–36.0%) and SSC (30.9–31.4%) (Table 1, Fig. 2). However, the GC content of *L. angelicifolium* (40.8%) was significantly lower than other species, which may be caused by the longest IR region (Figs. 1, 2).

**Table 1:** Plastome features of 26 *Ligusticum* accessions in this study

Species	Length (bp)				Gene number				GC content (%)			
	Total	LSC	SSC	IR	Total	Protein-coding	tRNA	rRNA	Total	LSC	SSC	IR
*L. capillaceum* (= *Ligusticopsis capillacea*)	147,808	91,907	17,503	19,199	129	85 (5)	36 (6)	8 (4)	37.5	36	31	44.1
*L. delavayi*	155,623	85,066	16,741	26,908	133	88 (8)	37 (7)	8 (4)	37.6	35.7	31	42.5
*L. hispidum* (= *Ligusticopsis hispida*)	147,797	91,846	17,627	19,162	129	85 (5)	36 (6)	8 (4)	37.4	35.9	30.9	44.1
*L. involucratum* (= *Ligusticopsis involucrata*)	147,752	91,782	17,560	19,205	129	85 (5)	36 (6)	8 (4)	37.4	35.9	30.9	44
*L. likiangense* (= *Ligusticopsis integrifolia*)	148,196	92,305	17,575	19,158	129	85 (5)	36 (6)	8 (4)	37.5	35.9	31	44.1
*L. pteridophyllum*	146,443	92,598	17,513	18,166	129	85 (5)	36 (6)	8 (4)	37.5	35.9	31.2	44.8
*L. scapiforme* (= *Ligusticopsis scapiformis*)	148,107	92,214	17,581	19,156	129	85 (5)	36 (6)	8 (4)	37.5	36	31	44.1
*L. thomsonii*	147,462	93,363	17,591	18,254	129	85 (5)	36 (6)	8 (4)	37.6	36	31.1	44.8
*L. sinense*_1	148,430	93,887	17,607	18,468	129	85 (5)	36 (6)	8 (4)	37.6	36	31.1	44.8
*L. sinense*_2	148,515	93,978	17,607	18,465	128	84 (4)	36 (6)	8 (4)	37.6	36	31.1	44.8
*L. jeholense*	148,493	93,932	17,629	18,468	128	84 (4)	36 (6)	8 (4)	37.6	36	31.1	44.8
*L. tenuissimum*	158,500	84,875	17,661	27,982	134	89 (9)	37 (7)	8 (4)	37.6	35.7	31.1	42.5
***L. oliverianum*_SP (= *Ligusticopsis oliveriana*)**	148,175	92,273	17,534	19,184	129	85 (5)	36 (6)	8 (4)	37.5	35.9	31	44.2
***L. oliverianum*_WC (= *Ligusticopsis oliveriana*)**	148,378	92,262	17,558	19,279	129	85 (5)	36 (6)	8 (4)	37.5	36	31	44.1
***L. daucoides* (= *Ligusticopsis daucoides*)**	148,078	91,666	17,582	19,415	129	85 (5)	36 (6)	8 (4)	37.4	36	30.9	43.9
***L. nematophyllum***	148,608	94,005	17,635	18,484	129	85 (5)	36 (6)	8 (4)	37.6	36	31.1	44.8
***L. brachylobum *(= *Ligusticopsis brachyloba*)**	148,163	92,273	17,556	19,167	129	85 (5)	36 (6)	8 (4)	37.5	36	31	44.1
***L. jeholense_*YX**	148,519	93,940	17,639	18,470	129	85 (5)	36 (6)	8 (4)	37.6	36	31.1	44.8
***L. angelicifolium***	163,802	76,900	17,464	34,719	145	100 (20)	37 (7)	8 (4)	37.4	35.8	31	40.8
***L. likiangense*_EY *(*= *Ligusticopsis integrifolia)***	148,025	92,196	17,589	19,120	129	85 (5)	36 (6)	8 (4)	37.5	36	31	44.2
***L. thomsonii_*MQ**	147,528	93,345	17,593	18,295	129	85 (5)	36 (6)	8 (4)	37.6	36	31.1	44.8
***L. involucratum*_DL (= *Ligusticopsis involucrata*)**	148,185	92,232	17,665	19,144	129	85 (5)	36 (6)	8 (4)	37.5	35.9	31	44.1
***L. pteridophyllum_*DL**	146,719	94,079	17,522	17,559	129	85 (5)	36 (6)	8 (4)	37.5	35.9	31.2	45
***L. weberbauerianum* (= *Hansenia weberbaueriana*)**	157,040	86,301	17,825	26,457	133	88 (8)	37 (7)	8 (4)	37.6	35.8	31.4	42.8
***L. litangense***	156,918	86,210	17,792	26,458	133	88 (8)	37 (7)	8 (4)	37.6	35.7	31.3	42.8
***L. tachiroei***	155,455	85,572	17,193	26,345	133	88 (8)	37 (7)	8 (4)	37.6	35.7	31.3	42.8

The 14 newly obtained plastome sequences are highlighted in bold

LSC, large single-copy; SSC, small single-copy; IR, inverted repeat

### Molecular evolutionary pattern of plastid protein-coding genes

**Fig. 3:**
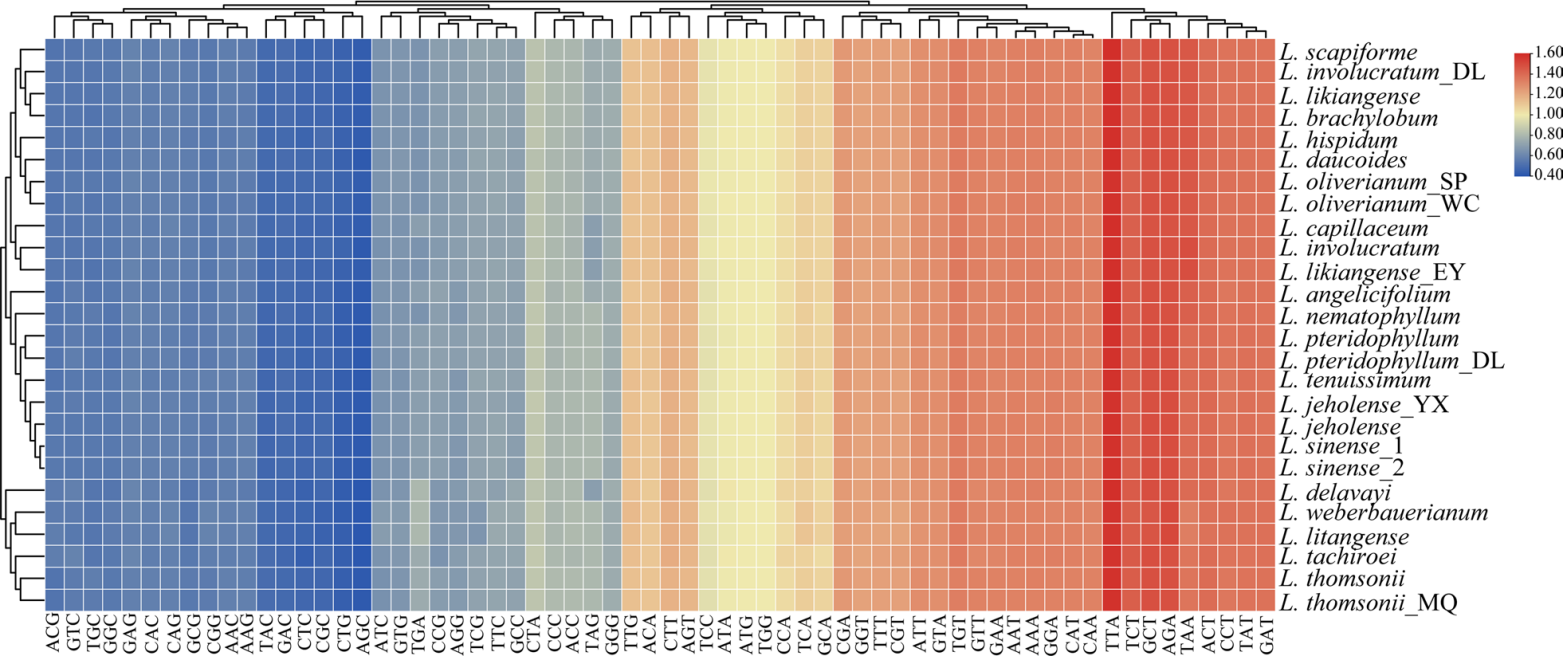
The RSCU values of 53 merged protein-coding sequences for 26 *Ligusticum* plastomes. Color key: the red values indicate higher RSCU values and the blue values indicate lower RSCU values

Fifty-three protein-coding sequences (CDSs) of each *Ligusticum* species were selected to determine the codon usage patterns. Codon usage bias was similar across all *Ligusticum* species (Additional file 4: Table S2, Fig. 3). We found that 2208–2246 codons (10.4–10.6%) encode Leucine, and 210–222 codons (1.0–1.1%) encode Cysteine, which were the most prevalent and rarest amino acids, respectively. Figure 3 demonstrates that about half of the codons were used more frequently. Specifically, 30 codons were used frequently with RSCU > 1, and all biased codons ended with a purine (A/T) except for TTG (Fig. 3). Within the 53 CDSs, the first and second codon positions had much higher GC content (45.9–46.0% and 38.1–38.3%) than the third codon positions (29.6–30.0%) (Additional file 4: Table S2). We identified 55–60 RNA editing sites for 20–23 protein-coding genes from each *Ligusticum* species. (Additional file 5: Table S3). Further analysis found that most RNA editing events occurred in the *ndh* gene (22–24). Although *Ligusticum* appeared to have a similar pattern of RNA editing, several specific editing sites have been picked out: *petD* (1 site; only identified in *L. involucratum*) and *rps8* (1 site; only identified in *L. jeholense*).

**Fig. 4:**
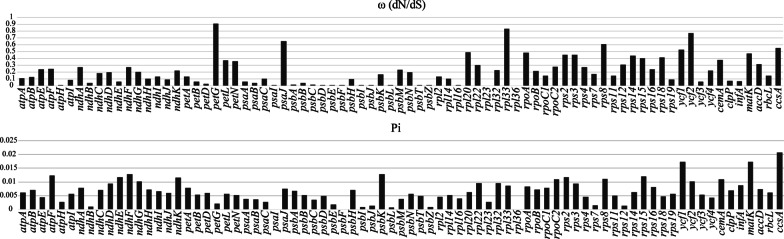
The dN/dS (ω) and nucleotide diversity (Pi) of the 79 protein-coding sequences within 26 *Ligusticum* plastomes

The ω values of CDSs for 79 plastid protein-coding genes ranged from 0.0001 to 0.9065 (Fig. 4), suggesting conservation of plastid protein-coding genes in *Ligusticum*. Most genes were under strong purifying selection with a very low ω value (ω < 0.5), yet the ω values in the range of 0.5 to 1.0 (indicating relaxed selection) were observed for seven genes *petG*, *ccsA*, *rps8*, *rpl33*, *psaJ*, *ycf1*, and *ycf2* (Fig. 4). However, we found that only three genes (*rps8*, *ycf1*, and *ycf2*) were under relaxed selection due to their significance (P < 0.05) after the likelihood ratio test (LRT) (Additional file 6: Table S4). Nucleotide diversity (Pi) of these 79 CDSs was calculated to assess the sequence divergence level (Additional file 6: Table S4). Among these, Pi values ranged from 0 to 0.02071 (Fig. 4). Six CDSs had relatively higher Pi values, including *matK*, *cemA*, *ycf1*, *psbK*, *ndhF*, and *atpF* genes, which revealed that these genes were more divergent and evolving more rapidly than other genes (Fig. 4). Conversely, CDS of *rpl36*, *psbF*, *psaI*, *psbL*, *psbI*, and *psbZ* genes shared very low Pi values, suggesting that these genes are highly conserved (Fig. 4). Collectively, the low Pi values also indicated that the plastid protein-coding genes were conserved in *Ligusticum*.

### Phylogenetic relationships

**Fig. 5:**
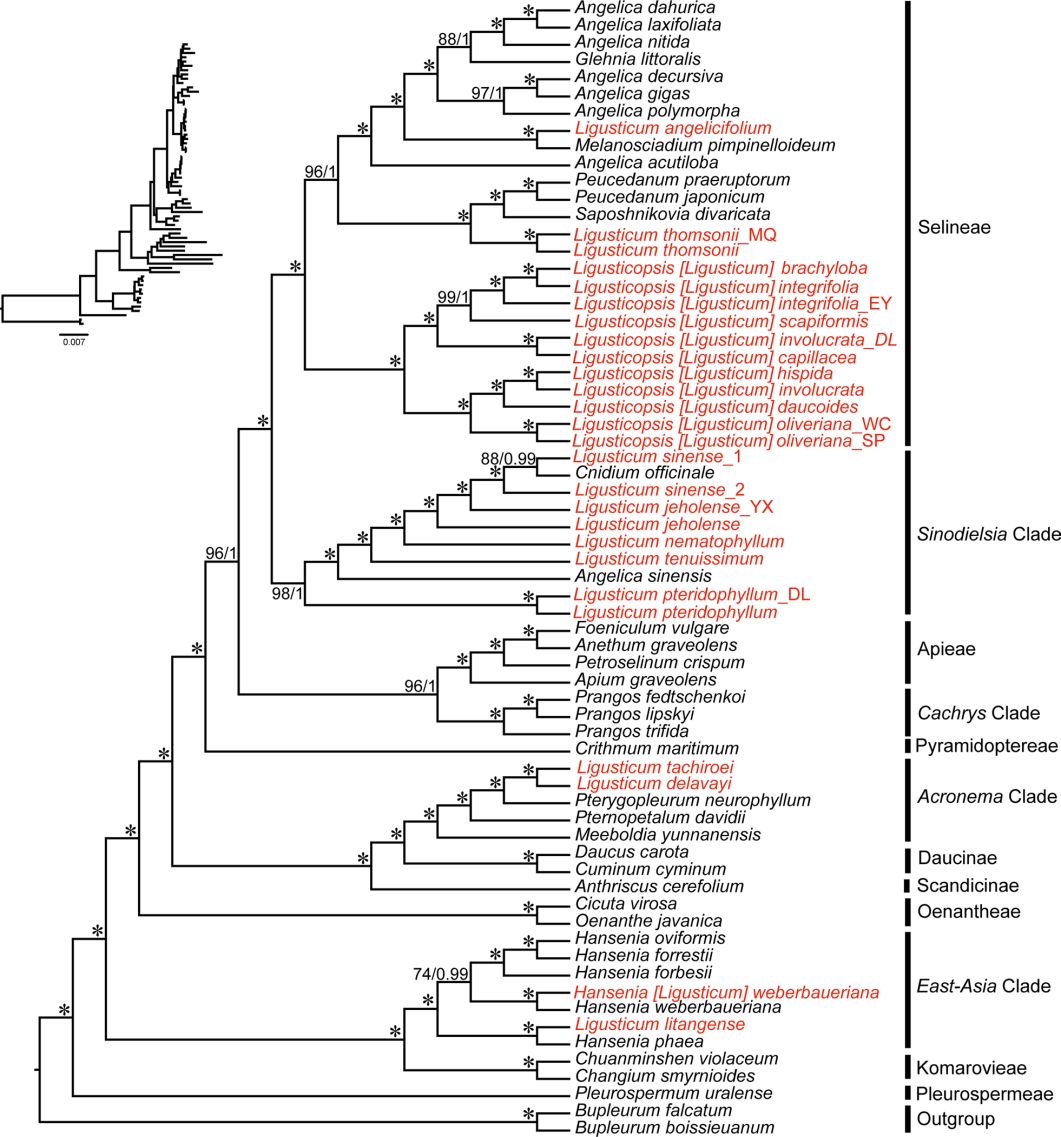
Phylogenetic relationships inferred from Maximum likelihood (ML) and Bayesian inference (BI) analyses based on 66 complete plastomes within Apiaceae. The bootstrap support values (BS) and posterior probabilities (PP) are listed at each node

**Fig. 6:**
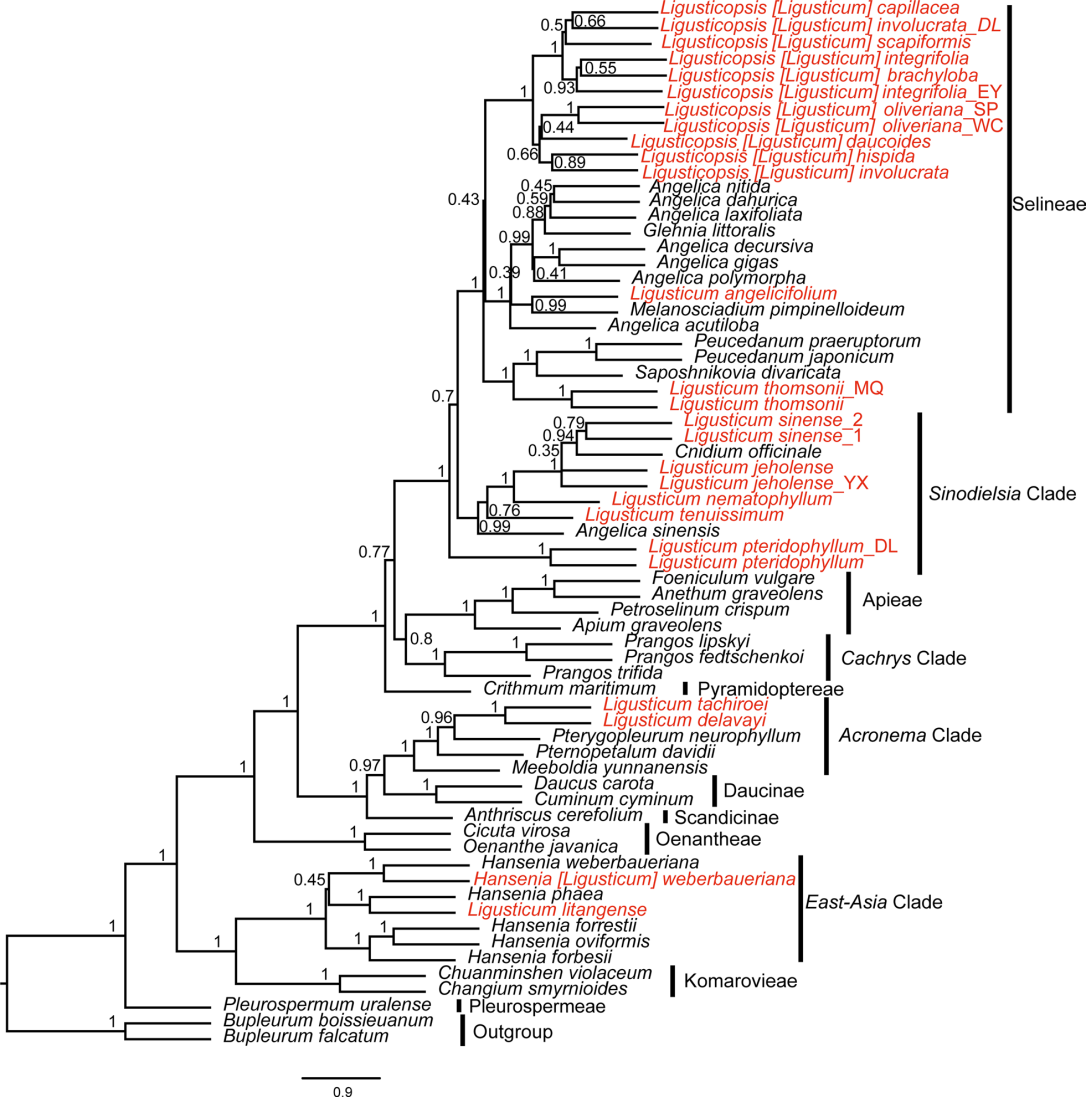
Phylogenetic relationships of 66 Apiaceae species inferred from 76 common protein-coding sequences based on the coalescent-based approach using ASTRAL. The local posterior probabilities (LPP) are listed at each node

We performed a series of phylogenetic analyses using two datasets (complete plastome sequences and 76 common CDSs) and two methods (concatenation and coalescent-based analyses) for 66 species of Apiaceae (Additional file 7: Table S5). The aligned two datasets were 124,230 bp and 65,979 bp long, with 23,598 and 8932 variable sites, and proportions of 18.99% and 13.54%, respectively. As expected, our analyses obtained robust support at most nodes. All phylogenetic analyses produced largely identical tree topologies, the incongruence mainly occurred in the interspecific relationships within clades, and the relationship between the clades was congruent except for the systematic position of *Cachrys* Clade (Figs. 5, 6; Additional file 2: Fig. S2). *Cachrys* Clade was resolved as sister to *Sinodielsia* Clade + Selineae ((Selineae, *Sinodielsia* Clade), *Cachrys* Clade) in the ML tree based on dataset-2 (BS = 54), while it was sister to Apieae in the other four phylogenetic trees with moderate-to-high support (Figs. 5, 6; Additional file 2: Fig. S2). For *Ligusticum*, it was still a non-monophyletic taxon, and the clades of these species were consistent with previous studies. Although we have enriched the plastome data of *Ligusticum*, the systematic position of *L. pteridophyllum* is still unclear in this study. *L. pteridophyllum* belonged to *Sinodielsia* Clade based on dataset-1 (BS = 98, PP = 1), while it was resolved as sister to *Sinodielsia* Clade + Selineae ((Selineae, *Sinodielsia* Clade), (*L. pteridophyllum, L. pteridophyllum_*DL)) in the other three phylogenetic trees (BS = 100, PP = 1, LPP = 1) (Fig. 6, Additional file 2: Fig. S2). Given that the variation level of the 76 CDSs and the incongruent topologies of dataset-2 (76 CDSs) obtained by different analyses (ML and BI), as well as the positions of *L. pteridophyllum* and *Cachrys* Clade were distinct from dataset-1, we then used 76 CDSs to perform a phylogenetic analysis according to the multi-species coalescent model by ASTRAL v5.7.3 [45] (Fig. 6). Thus, we used this coalescent-based phylogeny and concatenation-based phylogeny (dataset-1) as the basis in this study (Figs. 5, 6).

Compared to the results from concatenation-based phylogeny (dataset-1), the phylogenetic relationship among the clades in the coalescent-based phylogeny was identical, but the interspecific relationships within clades had a few discrepancies (Figs. 5, 6). The 26 *Ligusticum* accessions were distributed in four clades (*Acronema* Clade, Selineae, *Sinodielsia* Clade, and *East-Asia* Clade) (Figs. 5, 6). Two species (*L. tachiroei* and *L. delavayi*) fell into *Acronema* Clade and formed a clade with strong supports (BS = 100, PP = 1, LPP = 1). *L. weberbauerianum* (= *H. weberbaueriana*) and *L. litangense* fell into *East-Asia* Clade (BS = 100, PP = 1, LPP = 1). The *Sinodielsia* Clade is more complicated. In the concatenation-based phylogeny, eight *Ligusticum* accessions clustered with *C. officinale* and *A. sinensis* fell into *Sinodielsia* Clade with high support (BS = 98, PP = 1). However, *Sinodielsia* Clade was not recovered as a monophyletic group, because two *L. pteridophyllum* accessions were resolved as sister to *Sinodielsia* Clade + Selineae ((Selineae, *Sinodielsia* Clade), (*L. pteridophyllum, L. pteridophyllum_*DL)) in the coalescent-based phylogeny (LPP = 1). Most *Ligusticum* accessions were within Selineae, while they did not form a clade. Two *L. thomsonii* accessions clustered with *S. divaricate*, *P. praeruptorum*, and *P. japonicum*, and *L. angelicifolium* clustered with *M. pimpinelloideum*. Eleven other *Ligusticum* accessions formed a clade with strong support (BS = 100, PP = 1, LPP = 1).

## Discussion

### The diversity of plastome characteristics

By combining the 14 newly sequenced plastome sequences with the 12 published sequences of *Ligusticum*, we can represent all four currently identified clades in China. The *Ligusticum* plastomes were variable among the four clades, as well as the plastomes of Selineae and *Sinodielsia* Clade were significantly different from that of *Acronema* Clade and *East-Asia* Clade, which might have phylogenetic and taxonomic significance. Previous studies concluded that the total length of angiosperm plastomes is usually influenced by the contraction and expansion of the IRs [31, 32]. Similarly, we noticed that the longer total lengths of six *Ligusticum* (*L. angelicifolium*, *L. tenuissimum*, *L. litangense*, *L. weberbauerianum*, *L. delavayi,* and *L. tachiroei*) plastomes were determined by the expansion of IRs. We detected four types of LSC/IRb border (*ycf2*, *petB*, *rpl22*, and *rps19* genes) and four types of LSC/IRa border (*petB-trnH-GUG*, *trnL-CAA-trnH-GUG, rps19-trnH-GUG*, and *rpl2-trnH-GUG*), which were also reported in Apiaceae and other plant lineages [25, 28, 33–35]. Compared to the dynamically shifted LSC/IR border, the SSC/IR border was more conserved, as most SSC/IR borders were *ycf1* genes with a few exceptions in the 26 *Ligusticum* plastomes. In addition to IR border shifts, IR has been significantly increased, reduced, or even eliminated, such as in *Pelargonium* × *hortorum* [36], *Cephalotaxus oliveri* [37], some species of *Erodium* and Pinaceae [38, 39]. Gene content of *Ligusticum* was not conserved, mainly due to the increase of gene number caused by the expansion of IRs [40]. For example, *L. angelicifolium* possessed the most genes. The *rpl22*, *rps3*, *rpl16*, *rpl14*, *rps8*, *infA*, *rpl36*, *rps11*, *rpoA,* and *petD* genes located in LSC regions of other *Ligusticum* species, have moved to IR regions to become double-copy genes in *L. angelicifolium*. GC content of *Ligusticum* plastomes was close to other Apiaceae [25, 41]. High GC content was observed in IRs, which is probably due to the presence of the four rRNA genes [42, 43] as they had a GC content of up to 54.9–55.3% (Fig. 2).

### The evolutionary conservation of plastid protein-coding genes

Codon usage bias is an important evolutionary feature in the genome that can be influenced by many evolutionary processes [44]. Therefore, codon usage bias provides useful information for studying molecular evolution. GC content is generally the product of directional mutation pressure and is a critical factor affecting codon usage [38, 44, 45]. All 26 *Ligusticum* plastomes had a strong bias toward A/T at the third codon position as observed in other angiosperm species [46, 47]. High AT content in plastomes is the major reason for bias codons ending with A/T [48]. RNA editing is one of the posttranscriptional maturation processes of primary transcripts, which allows nucleotide insertion/deletion and conversion to alter transcripts [49, 50]. The first chloroplast RNA editing was discovered in maize *rpl2* transcript, in which an initiation codon ACG changes to AUG [51]. After that, RNA editing has been found in a growing number of higher plant chloroplasts. The *ndh* genes encode subunits of the plastid NDH (NADH dehydrogenase-like) complex, which contained most RNA editing sites for *Ligusticum* species. The *ndh* genes play an important role in mediating cyclic electron flow around photosystem I and facilitating chlororespiration [52]. Therefore, RNA editing on the *ndh* genes is more likely to ensure the physiological and biochemical processes of the plant. Similar codon usage and RNA editing patterns for 26 *Ligusticum* plastomes possibly because of the evolutionary conservation of plastomes among angiosperms.

The synonymous and non-synonymous nucleotide substitution pattern is a major indicator in the study of gene evolution. The ratio (ω) of dN/dS is generally interpreted as: purifying selection (ω < 1, especially less than 0.5), positive selection (ω > 1), neutral evolution (ω = 1), whereas ω value close to 1 indicates relaxed selection [53, 54]. All protein-coding genes held low ω and Pi values, suggesting the conservation of plastid genes in *Ligusticum*. Three genes (*rps8*, *ycf1*, and *ycf2*) were under relaxed selection. The *rps8* is one of the genes that encodes a protein for the small ribosomal subunits, therefore essential for the plastid ribosome [55]. The *rps8* gene was under positive selection in *Curcuma* [56]. The plastid gene *rps8* RNA editing defect accounted for the low-temperature sensitivity in rice and maize [57, 58]. This indicated that *rps8* gene is very important for plant adaptability. The *ycf1* gene, the second-largest gene in the plastome, is indispensable for photosynthetic protein import and is therefore vital for plant viability [59]. Positive selection or relaxed selection on *ycf1* have been observed in *Bulbophyllum* [60] and Lennoaceae [61]. *ycf2* is a conserved open reading frame with the exact function still unknown, although its putative gene product is a protein of 2280 amino acids [55, 62]. *Ligusticum* is mainly distributed in the alpine and subalpine regions of Southwest China. Consequently, we speculated that the possible relaxed selection pressure on these three genes may be related to adapting to high-altitude living environments.

### Phylogenetic relationships and taxonomic implications

Early studies have shown that the genus *Ligusticum* was a non-monophyletic group [14–21], and was divided into six clades: *Acronema* Clade, *Conioselinum chinense* Clade, Pyramidoptereae, Selineae, *Sinodielsia* Clade, and *East-Asia* Clade [8]. Here, the *Ligusticum* plastomes of 20 species (26 accessions) representing all four currently recognized clades in China were used to reconstruct the phylogenetic trees. We used different datasets and methods to perform phylogenetic analyses to obtain robust phylogenetic relationships of this genus, which revealed that the plastome‐scale data is a promising tool for resolving the phylogeny of the controversial taxon. In the concatenation-based phylogeny analysis, dataset-1 and dataset-2 yielding slightly different topologies may be primarily due to the discrepancy in the number of variable sites. On consideration, we finally decided to use coalescent-based phylogeny and concatenation-based phylogeny (dataset-1) as the basis to explore the phylogeny of *Ligusticum*.

Among the four clades of *Ligusticum*, only *Sinodielsia* Clade was not monophyletic, owing to the two accessions of *L. pteridophyllum* not being clustered with other *Sinodielsia* Clade species in the coalescent-based result. This was also observed in previous studies [16]. In fact, the systematic position of *L. pteridophyllum* has not been correctly described. *L. pteridophyllum* was once placed in the Selineae [14] or *Sinodielsia* Clade [8]. Zhou et al. [8] involved more *Ligusticum* species in their study, and the results were more reliable. Morphologically, *L. pteridophyllum* does not share several general characteristics of *Ligusticum* in Selineae, such as pinnate bracteoles, and with fibrous remnant sheaths at the stem bases (Additional file 8: Table S6). Taken together, we agreed that *L. pteridophyllum* belongs to *Sinodielsia* Clade, while more species in *Sinodielsia* Clade should be involved to verify this result. Six other accessions of *Ligusticum* clustered with *C. officinale* and formed a clade in *Sinodielsia* Clade, with *C. officinale* being closely related to *L. sinense*. *C. officinale* has been referred to as *L. officinale* [8], and the strong cross-hybridization of genomes was found between *C. officinale* and *L. sinense* [63].

Selineae contained most *Ligusticum* species, whereas they did not group together in this tribe. Two accessions of *L. thomsonii* and *L. angelicifolium* were not clustered with eleven other accessions of *Ligusticum*, which may be explained by morphology. The most obvious difference among them is bracteole and fruit. *L. thomsonii* and *L. angelicifolium* have linear or lanceolate bracteoles, as well as the prominent dorsal and intermediate ribs, winged lateral ribs. However, the eleven other accessions of *Ligusticum* have pinnate bracteoles, as well as raised dorsal and intermediate ribs, winged lateral ribs (Additional file 8: Table S6). The genus *Ligusticopsis* with 14 species was separated from *Ligusticum* because of its prominent calyx teeth [7], however, these 14 species did not form a monophyletic group. Pimenov [64] proposed several new nomenclarural combinations in *Ligusticopsis*, which included seven species (*L. brachylobum, L. capillaceum, L. daucoides, L. hispidum, L. involucratum, L. likiangense,* and *L. scapiforme*) analysed in this study. We approved this conclusion and suggested that *L. oliverianum* should be also incorporated into the genus *Ligusticopsis* based on molecular and morphological evidence (Figs. 5, 6; Additional file 2: Fig. S2; Additional file 8: Table S6).

*L. weberbauerianum* (= *H. weberbaueriana*) and *L. litangense* fell into *East-Asia* Clade. The two accessions of *H. weberbaueriana* (= *L. weberbauerianum*) were sisters in the phylogenetic trees, thus we agreed with this treatment that *L. weberbauerianum* is a synonym of *H. weberbaueriana* [65, 66]. Several studies have shown that *L. litangense* should be placed in *Hansenia* rather than *Ligusticum* [64]. *L. litangense* was related to *H. phaea* within *Hansenia*, which implied that *L. litangense* should be merged into *Hansenia* [8, 64, 67].

Two species (*L. tachiroei* and *L. delavayi*) that were in *Acronema* Clade formed a clade with strong support. However, the two species should be transferred from *Ligusticum* to another genus according to prior research [64]. It is worth mentioning that the generic type of *Ligusticum* (namely, *Ligusticum scoticum*) was placed in *Acronema* Clade [8, 14]. In the present study, most of the *Ligusticum* species were not fell into *Acronema* Clade, except for *L. tachiroei* and *L. delavayi*. Moreover, Zhou et al. [8] found that *L. scoticum* and *L. scoticum* subsp. *hultenii* occurred in *Acronema* Clade formed a monophyletic group with high support, and they were separated from *L. tachiroei* and *L. delavayi*. Consequently, in the light of the plastome’s results, we concluded that the current circumscription of the Chinese *Ligusticum* should be reduced, which is consistent with Zhou et al.’s [8] study based on ITS sequences.

*Ligusticum* is one of the most taxonomically difficult taxa within Apiaceae, largely due to the diversity of flowers, leaves, bracteoles, and mericarps. *Ligusticum* is described as a dustbin genus, as it contains several species that cannot be classified correctly [8]. In addition, fruit is the most important taxonomic character of *Ligusticum*, yet most species of the genus grow at high elevations with late fruiting. As a perennial herb, the genus sometimes does not blossom and bear fruit in a year, which was encountered many times during our field sampling. These factors make it difficult to sample the fruits of *Ligusticum*, resulting in a lack of unique taxonomic characters. Thus, the fruits of *Ligusticum* should also be collected to provide a morphological foundation for the taxonomic revision of *Ligusticum*. Together with the molecular phylogenetic analyses, including the use of traditional molecular markers and plastome-scale data [8, 14–21], we therefore strongly argue that a revision of *Ligusticum* taxonomy is necessary. Further studies will require more taxa of *Ligusticum* and its allied genera, as well as combine molecular and morphological evidences to resolve the taxonomy and delimitation of *Ligusticum*. Overall, our study provides new insights into the taxonomic classification of *Ligusticum* and will serve as a framework for future studies on the taxonomy and delimitation of *Ligusticum* from the perspective of the plastid genome.

## Materials and methods

### Taxon sampling and DNA extraction

We newly sequenced 14 plastomes, including 13 species covering four clades of *Ligusticum*. We also recovered 12 plastomes from the NCBI database (https://www.ncbi.nlm.nih.gov/). In total, we sampled 20 species (26 accessions) within *Ligusticum* (Additional file 3: Table S1). Fresh leaves from adult plants of each newly sequenced species were collected in the field and immediately dried with silica gel for future DNA extraction. These plants are not protected, therefore permission is not required for sample collection. The species identification of the plant material was undertaken by Xingjin He (Sichuan University, Chengdu, China). Voucher specimens were deposited at the herbarium of Sichuan University (Chengdu, China) (Additional file 3: Table S1). Total genomic DNA was extracted from silica-dried leaves with a CTAB protocol [68]. The quality and concentration of the DNA products were assessed using 1% agarose gel electrophoresis and a Quant-iT PicoGreen dsDNA Assay Kit.

### Illumina sequencing, assembly, and annotation

The DNA library with an insert size of 400 bp was constructed using the TruSeq DNA Sample Preparation Kits (Illumina) according to the manufacturer’s protocol. The DNA library was sequenced using Illumina NovaSeq platform with an average paired-end read length of 150 bp at Shanghai Personal Biotechnology Co., Ltd. (Shanghai, China). The quality of the newly generated sequencing data was assessed using the FastQC v0.11.9 software [69]. The obtained raw reads were adapter-trimmed and quality-filtered by AdapterRemoval v2 (trimwindows = 5 and minlength = 50) [70], yielding at least 5 GB clean reads for each species. Clean reads were then used to perform a de novo assembly by NOVOPlasty v2.6.2 (K-mer = 39) [71]. The seed sequence was the *rbcL* gene from the reference plastome sequence of *L. delavayi* (NC_049052) [16]. The annotation of the 14 plastomes was completed using GeSeq [72], and we manually adjusted the positions of start and stop codons and the exon/intron boundaries in Geneious v9.0.2 (Biomatters Ltd., Auckland, New Zealand) against its congeneric species. The 14 newly obtained plastome sequences are available at the GenBank (Accession numbers: MZ532560–MZ532573). The circle plastome map was generated using the online program OrganellarGenomeDRAW (OGDRAW) [73].

### Molecular evolutionary analysis

To identify the codon usage patterns, MEGA6 [74] was employed for the codon usage bias analyses using protein-coding genes with CDS lengths greater than 300 bp to avoid sampling bias [75]. The heatmap was drawn using TBtools [76]. The total GC content and the GC content for the first, second, and third codon positions of these CDSs were also calculated by MEGA6 [74]. To reveal the composition and characteristics of RNA editing, the potential RNA editing sites in protein-coding genes of 26 *Ligusticum* plastomes were predicted using the PREP-Cp program [77] with a cutoff value of 0.8.

To explore the selection patterns on the plastid protein-coding genes, the nonsynonymous (Ka) and synonymous (Ks) nucleotide substitution rates of 79 protein-coding genes were calculated using a site-specific model implemented in Codeml program (seqtype = 1, model = 0, NSsites = 0, 1, 2, 3, 7, 8) [78] of PAML4.9 software [79]. Codon frequencies were determined using the F3 × 4 model and gapped regions were excluded with the parameter “cleandata = 1” option. For PAML analyses, the ML tree constructed using RAxML v8.2.8 [80] based on 79 plastid protein-coding genes was used as the input treefile. Likelihood ratio test (LRT) with a Chi-square distribution was used to confirm the model fit. The Bayes Empirical Bayes (BEB) analysis was used to statistically identify selected sites with posterior probabilities ≥ 95%. The nucleotide diversity (Pi) of the CDSs of 79 protein-coding genes was also calculated using DnaSP v5.1 [81].

### Phylogenetic analysis

Sixty-six species of Apiaceae were used to infer the phylogeny of *Ligusticum*, among which, two *Bupleurum* species served as the outgroups (Additional file 7: Table S5). Both concatenation and coalescent-based analyses were carried out. For the concatenation-based approach, two datasets were used to conduct the phylogenetic analysis: dataset-1 was the complete plastomes (excluding one inverted repeat region); dataset-2 encompassed the 76 common protein-coding sequences (CDSs) (Genes list used in the phylogenetic analyses was provided in Additional file 7: Table S5). The number of variable sites of the two datasets was calculated by MEGA6 [74]. To avoid duplicate regions increasing the phylogenetic signal, the second IR was removed from the first dataset. Sequence alignment was achieved using the MAFFT v7.221 [82] and ambiguously aligned areas were removed using Gblocks v0.91b [83] with the default setting. The nucleotide sequences of the 76 common CDSs were extracted and then concatenated into a supermatrix using PhyloSuite v1.2.1 [84]. The maximum likelihood (ML) analysis was conducted in RAxML v8.2.8 [80] with 1000 bootstrap replicates and GTRGAMMA model. Bayesian inference (BI) was carried out using MrBayes v3.1.2 [85] with the best-fitting evolutionary model determined by Modeltest v3.7 [86]. The selected models for complete plastomes and 76 common CDSs in BI analyses were TVM + I + G and GTR + I + G, respectively. Markov chain Monte Carlo (MCMC) algorithm was run for 5,000,000 generations, with one tree sampled every 1000 generations. The MCMC reached stationarity when the average standard deviation of the split frequencies was less than 0.01. The initial 25% of the sampled data was discarded as burn-in, and the consensus tree was generated using the remaining trees.

Given that the variation level of different genes (Additional file 7: Table S5), and to provide the best estimate of the phylogeny of *Ligusticum*, we also undertook a coalescent-based analysis using ASTRAL v5.7.3 [87]. This approach inferred a species tree using individual gene trees. The gene trees were separately generated for 76 CDSs using RAxML v8.2.8 [80] with 500 bootstraps and GTRGAMMA model. The 76 RAxML best ML gene trees were used as input for ASTRAL v5.7.3 [87] to estimate a species tree with local posterior probability (LPP) [88].

## Conclusions

In this study, we integrated 26 plastomes (including 14 newly sequenced plastomes) to perform molecular evolutionary analysis and phylogenetic reconstruction. These plastid genomes exhibited diverse plastome characteristics. The analyses of codon usage, RNA editing, dN/dS, and nucleotide variability (Pi), have demonstrated the conservation of the protein-coding genes in *Ligusticum*. The phylogenetic analyses obtained a more robust molecular phylogeny than prior studies and showed the non-monophyly of *Ligusticum* containing four clades. Our results emphasized that the current circumscription of the Chinese *Ligusticum* should be reduced. Wider taxon sampling including related species of *Ligusticum* will be necessary to explore the phylogenetic relationships of *Ligusticum*. Overall, our study provided new insights into the phylogenetic relationships of *Ligusticum* and would serve as a framework for the taxonomy and delimitation studies of this genus.

## Supplementary Information

**Additional file 1: Figure S1.** Gene map of *Ligusticum angelicifolium* plastomes. The genes shown outside of the circle are transcribed clockwise, while those inside are transcribed counterclockwise. The genes belonging to different functional groups are color-coded. The innermost darker gray represents the GC content of the plastome.

**Additional file 2: Figure S2.** Phylogenetic relationships of 66 Apiaceae species inferred from 76 common protein-coding sequences based on Maximum likelihood (ML) and Bayesian inference (BI) analyses. The bootstrap support values (BS) and posterior probabilities (PP) are listed at each node.

**Additional file 3: Table S1.** Information regarding the 26 *Ligusticum* accessions used in this study.

**Additional file 4: Table S2.** Codon and base compositions for protein-coding sequences of 53 plastid genes in the 26 *Ligusticum* plastomes.

**Additional file 5: Table S3.** RNA editing sites analyses of the 26 *Ligusticum* plastomes.

**Additional file 6: Table S4.** The ω (dN/dS) and Pi values for protein-coding sequences of 79 plastid genes in the 26 *Ligusticum* plastomes.

**Additional file 7: Table S5.** List of 66 species and 76 common protein-coding sequences used in the phylogenetic analyses.

**Additional file 8: Table S6.** Comparison of the morphology of 20 *Ligusticum* species.

## Data Availability

The newly sequenced plastid genome sequences were deposited into GenBank (MZ532560–MZ532573).

## Abbreviations

BEB: Bayes empirical Bayes
BI: Bayesian inference
bp: Base pair
BS: Branch support
CDS: Protein-coding sequences
CTAB: Cetyl trimethylammonium bromide
IR: Inverted repeat
LPP: Local posterior probability
LRT: Likelihood ratio test
LSC: Large single copy
MCMC: Markov chain Monte Carlo
ML: Maximum likelihood
PP: Posterior probability
rRNA: Ribosomal RNA
RSCU: Relative synonymous codon usage
SSC: Small single copy
tRNA: Transfer RNA
