# Supplementary figures and images for: Molecular evolution and phylogenetic relationships of Ligusticum (Apiaceae) inferred from the whole plastome sequences
Source: BMC Ecol Evol. 2022 Apr 30;22:55. doi: 10.1186/s12862-022-02010-z (PMC9063207; doi:10.1186/s12862-022-02010-z)

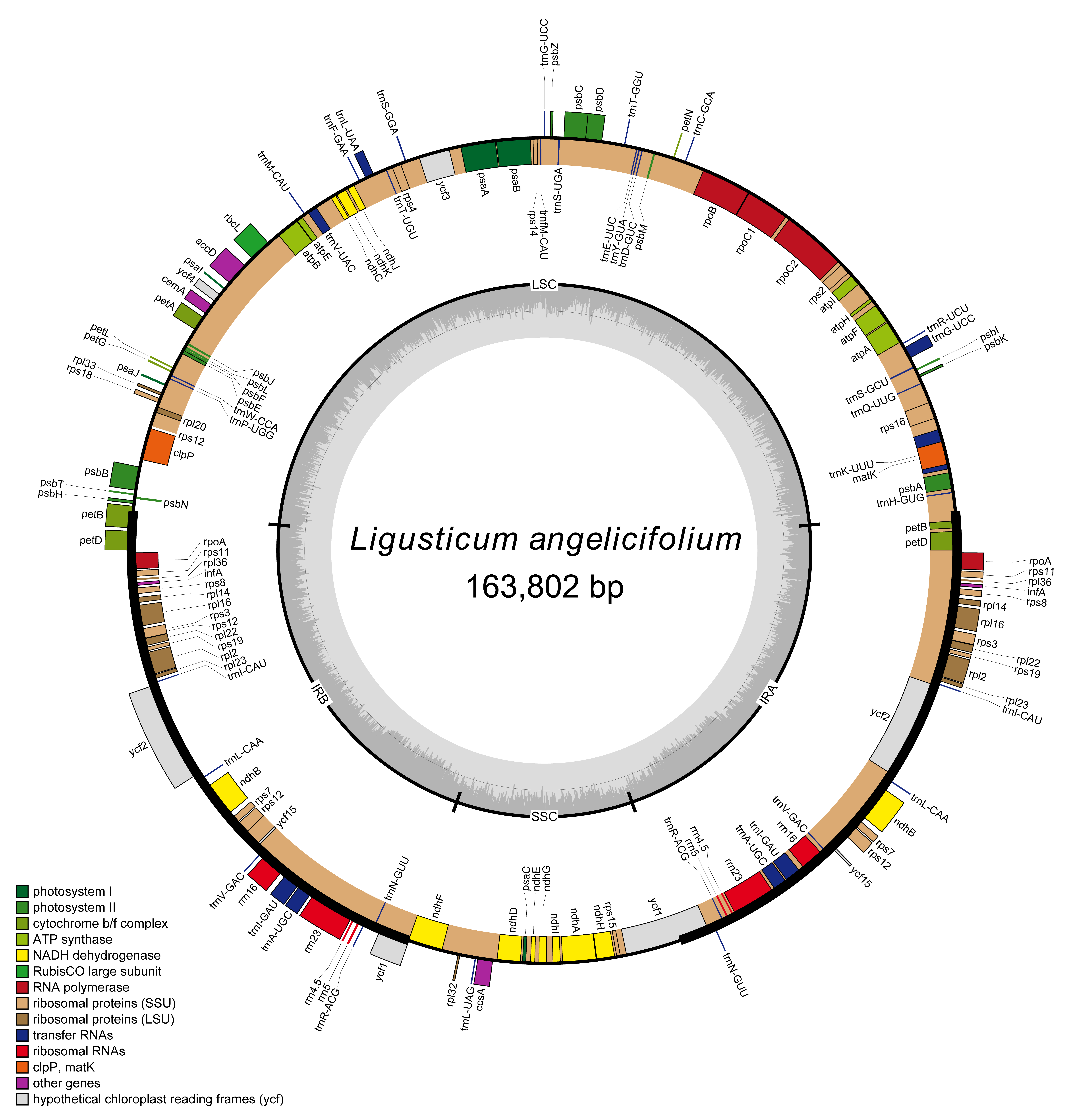

Supplement: Supplementary file 1 — Additional file 1: Figure S1. Gene map of Ligusticum angelicifolium plastomes. The genes shown outside of the circle are transcribed clockwise, while those inside are transcribed counterclockwise. The genes belonging to different functional groups are color-coded. The innermost darker gray represents the GC content of the plastome. [file 12862_2022_2010_MOESM1_ESM.tif]

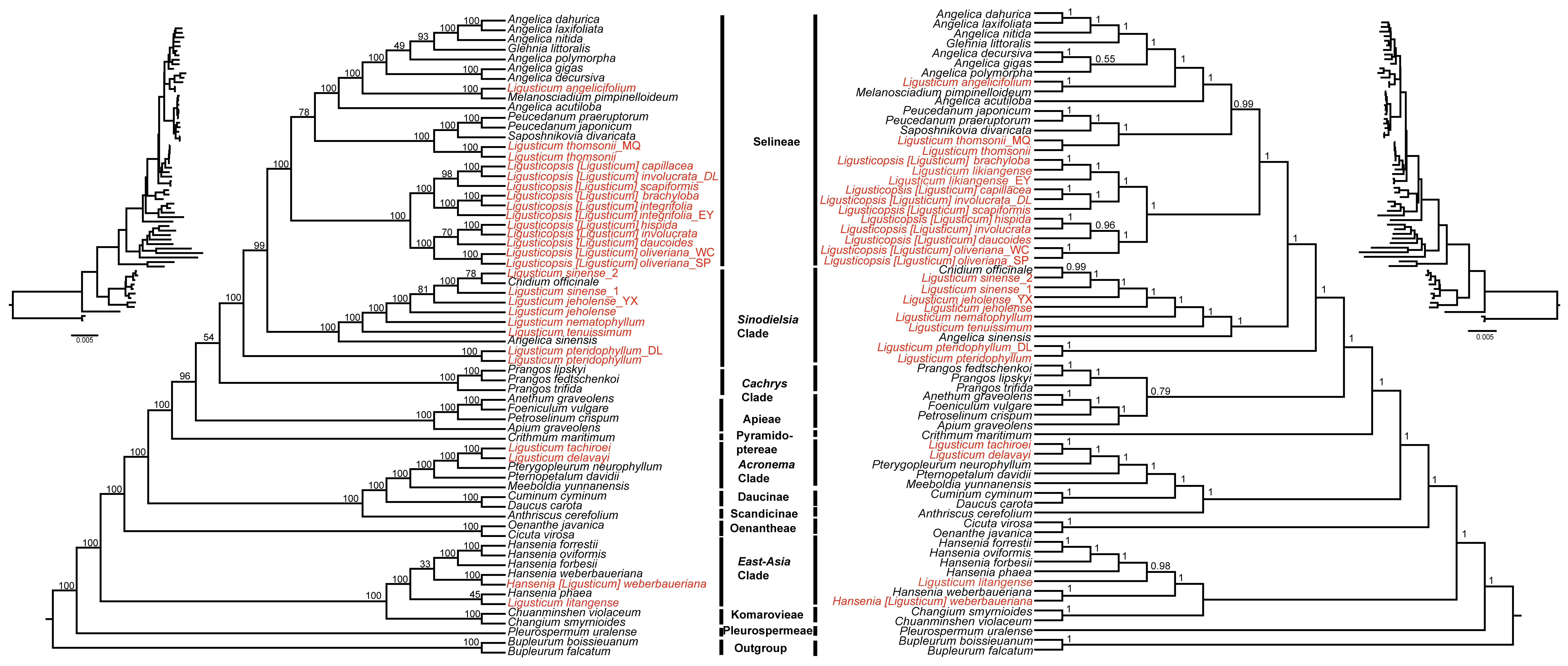

Supplement: Supplementary file 2 — Additional file 2: Figure S2. Phylogenetic relationships of 66 Apiaceae species inferred from 76 common protein-coding sequences based on Maximum likelihood (ML) and Bayesian inference (BI) analyses. The bootstrap support values (BS) and posterior probabilities (PP) are listed at each node. [file 12862_2022_2010_MOESM2_ESM.tif]
